# Black immigrants in São Paulo-Brazil: sociodemographic profile, reason for coming, embracement, and health

**DOI:** 10.1590/1980-220X-REEUSP-2022-0448en

**Published:** 2023-08-28

**Authors:** Maria Cecília Leite de Moraes, Linda Concita Nunes Araújo, Climene Laura de Camargo

**Affiliations:** 1 Universidade Federal da Bahia, Escola de Enfermagem, Grupo CRESCER, Salvador, BA, Brazil.

**Keywords:** Human Migration, Black People, Women’s Health, User Embracement, Migración Humana, Población Negra, Salud de la Mujer, Acogimiento, Migração Humana, População Negra, Saúde da Mulher, Acolhimento

## Abstract

**Objective:**

To understand the factors interfering in the living conditions (health) of black immigrant women. Therefore, we sought to know the sociodemographic profile, the reasons that led them to immigration, the embracement provided in the country, the presence of post-immigration illness, and the type of disease.

**Method:**

Quali-quantitative, cross-sectional study, carried out between March and October 2018, in the city of São Paulo-Brazil, with 33 black immigrant women. Data were obtained through interviews, guided by a semi-structured questionnaire. The responses were analyzed using the Discourse of the Collective Subject technique.

**Results:**

Findings showed that 69% of the immigrants studied came from Angola, 45% feel discriminated against, 45.6% report post-immigration illness, with emotional issues being highlighted.

**Conclusion:**

The need to organize an internal agenda to serve similar groups is acknowledged, an essential attribution to the nation that aims to receive people, a commitment that refers to the promotion of means to embrace, aggregate, and incorporate people as citizens.

## RESUMO

**Objetivo:**

Apreender os fatores que interferem nas condições de vida (saúde) de mulheres negras imigrantes. Para tanto, buscou-se conhecer o perfil sociodemográfico, os motivos que as levaram à imigração, o acolhimento recebido no país, a presença de adoecimento pós-imigração e a tipologia da doença.

**Método:**

Estudo qualiquantitativo, transversal, realizado no período entre março e outubro de 2018, no município de São Paulo-Brasil, com 33 mulheres negras imigrantes. Os dados foram obtidos por meio de entrevistas, norteadas por um questionário semiestruturado. As respostas foram analisadas atendendo à técnica do Discurso do Sujeito Coletivo.

**Resultados:**

Dentre os achados, observou-se que 69% das imigrantes estudadas vieram de Angola, 45% sentem-se discriminadas, 45,6% relatam adoecimento pós imigração, destacando-se as questões emocionais.

**Conclusão:**

Reconhece-se a necessidade de organizar uma agenda interna para atender a grupos semelhantes, atribuição essencial à nação que se propõe a receber, compromisso que remete à promoção de meios para acolher, agregar e incorporar as pessoas como cidadãs.

## RESUMEN

**Objetivo:**

Aprehender los factores que interfieren en las condiciones de vida (salud) de las mujeres negras inmigrantes. Para ello se buscó conocer el perfil sociodemográfico, los motivos que las llevaron a la inmigración, la acogida recibida en el país, la presencia de enfermedades post-inmigración y el tipo de enfermedad.

**Método:**

Estudio cuali-cuantitativo, transversal, realizado entre marzo y octubre de 2018, en la ciudad de São Paulo-Brasil, con 33 mujeres negras inmigrantes. Los datos fueron obtenidos a través de entrevistas, guiadas por un cuestionario semiestructurado. Las respuestas fueron analizadas mediante la técnica del Discurso del Sujeto Colectivo.

**Resultados:**

Entre los hallazgos, se observó que el 69% de los inmigrantes estudiados procedían de Angola, el 45% se siente discriminado, el 45,6% relata enfermedad post-migración, destacándose cuestiones emocionales.

**Conclusión:**

Se reconoce la necesidad de organizar una agenda interna para atender a grupos similares. Atribución esencial a la nación que se propone recibir, compromiso que se refiere a la promoción de medios para acoger, agregar e incorporar a las personas como ciudadanos.

## INTRODUCTION

Human movement has been inscribed in history since ancient times. The Bible, in Exodus, highlights the crossings as paths to salvation and freedom. Conceptually, migrations are human displacements often associated with economic factors, ethnic and religious conflicts^(1)^. Recently, the theme has been strengthened by the distinct contribution to new population formations in different countries.

The migratory agenda highlights a significant increase in the number of women, making the terms genderization or feminization, which mark the female presence in migrations, become relevant. This trend includes particularly African women from Angola and Cape Verde^(2)^.

Four terms are fundamental for understanding the migration process: migrant, immigrant, emigrant, and refugee. A migrant is someone who moves across a well-defined border. The immigrant crosses the border, enters another country to reside or work; the emigrant leaves his/her place of origin. The refugee is forced to emigrate due to persecution or threats to life motivated by ethnic-racial, religious, and/or political issues^(3)^.

In Brazil, the term immigrant is used for individuals from developing countries and has a negative undertone. The term foreigner characterizes individuals coming from developed countries, with relevant social and professional status^(4)^.

The arrival of an immigrant is usually followed by lack of information, lack of embracement, of humanized care, a status that refers to iniquity, inequality, and exclusion. Iniquity alludes to the idea of injustice; inequality marks the opportunity differentiated according to the individual’s social position. Exclusion is the disarticulation between the subject and the social group in which he/she is inserted, comprises non-participation in the full benefits of society^(4)^.

In Latin America, especially in Brazil, there is a scenario of social oppression. This circumstance arises with the violations experienced in the colonial period and, not infrequently, remains thus far^(5)^.

Migratory difficulties are accentuated by ethnic-racial differences. Racism intensifies tribulations, as the black migrant is an individual rejected by the Brazilian society^(6)^.

Given the aforementioned aspects, it is believed that intersectionality between being a black woman and an immigrant emerges as a significant contribution to research. Intersectionality encompasses the understanding of gender and its multiple and simultaneous relationships with inequality^(7)^. The study group lives the fullness of these intersections, which have a strong impact on their living conditions. Knowing them can contribute to effective inclusion actions. This article shows a study with people living in the city of São Paulo, who make up this community, showing sociodemographic profiles, displacement and embracement situation, health conditions, and project to stay in the country.

## METHOD

### Design of Study

This was a cross-sectional, exploratory and descriptive field study, with a quantitative and qualitative approach in the form of narrated and written responses.

### Local

The interviews took place in 06 (six) different locations, covering the north, south, east and center regions of the city of São Paulo. There were 04 (four) confessional establishments (religious nature), a Non-Governmental Organization (NGO), and an Occupation of Homeless Residents that shelters a group of immigrants.

### Population

Participants were 33 black women, recognized by the investigator’s subjectivity (phenotypic aspect) and subsequently self-declared as such (information filled in the demographic profile); immigrants, over 18 years of age, who understood and spoke Portuguese.

### Selection Criteria

The selection was made based on convenience criteria for the investigator.

### Data Collection

For the collection of information, a script organized into two sections was prepared: a questionnaire on the demographic data of the participants and an interview related to the migrant’s life circumstances: reason for immigration, reception in the country, health and migration process (presence of illness after immigration and typologies of the disease), and a project to stay in Brazil. All interviews were recorded. Data collection took place between March and October 2018.

### Data Analysis and Treatment

For the analysis of qualitative data, the Discourse of Collective Subject (DCS) Technique was used^(8)^. To comply with the technique, the answers were grouped according to the Central Ideas (CI), which are the frameworks of the Key Expressions (KE) narrated by the respondents. The procedure allows linking the KE, adding them, and originating the DCSs. The statements are written in the first person singular and include the manifestation of all participants. Furthermore, qualitative data are expressed in absolute and relative frequencies (percentages).

Quantitative data are described in means, standard deviation, minimum, and maximum values. The software used to make the calculations was SPSS, version 21. This data is displayed in tables and graphs.

### Ethical Aspects

For the identities of the participants to be preserved, each one of them was designated by a number, according to the order in which the interview was carried out (E1, E2....E33). All members signed the Free and Informed Consent Form before the start of data collection. The study complies with Resolution 466/12, having been approved under opinion 2518235, on 02/28/2018 by the Research Ethics Committee of the Public Health School of Universidade de São Paulo.

## RESULTS

Thirty-three black immigrant women, aged between 19 and 38 years (mean age 31.2 years, standard deviation 5.2 years) participated in the study. As for the country of origin, 23 women (69.7%) came from Angola, 5 from Haiti, 3 from Congo, and 2 from Ivory Coast. With regard to education level, 54.5% (18 women) finished high school, four had an undergraduate degree. The current activity is varied, and almost half of the interviewees (45.5%) were unemployed. Table 1 refers to the sociodemographic description of the interviewed immigrants.

**Table 1:** Sociodemographic profile of immigrants interviewed entrevistadas – São Paulo, SP, Brazil, 2018.

Variables	Statistics
**Age (years) - mean (sd)**	31.2 (5.2)
**Time in Brazil (months) - mean (sd)**	27.5 (20.7)
**Country of Origin - n(%)**	
Angola	23 (69.7%)
Haiti	5 (15.2%)
Congo	3 (9.1%)
Ivory Coast	2 (6.1%)
**Level of education - n(%)**	
Unfinished elementary school	1 (3.0%)
Finished elementary school	1 (3.0%)
Unfinished high school	4 (12.1%)
Finished High School	18 (54.5%)
Unfinished undergraduate degree	4 (12.1%)
Finished undergraduate degree	4 (12.1%)
Did not answer	1 (3.0%)
**Current Activity - n (%)**	
Unemployed	15 (45.5%)
Hairdresser	5 (15.2%)
Student	4 (12.1%)
Dressmaker	2 (6.1%)
Cleaning	2 (6.1%)
Health agent	1 (3.0%)
Activist/Journalist	1 (3.0%)
Freelance works (side hustle)	1 (3.0%)
Makeup artist	1 (3.0%)
Did not answer	1 (3.0%)

sd = standard deviation; n = absolute frequency, % = relative frequency.

Source: Research on Black Immigrant Women – São Paulo, 2018.

On the reason for immigration, 11 Central Ideas emerged: Economic issues (21.3%), War (15.2%), Violence (12.1%), Adventure (12.1%), Family problems (9.1%), Study and religion (9.1% ), Political issues (6.1%), Health (3.0%), Family decision (3.0%), Study (3.0%), Family reunification (3.0%), Did not answer (3.0%).

### Central Idea A - Economic Issues

**DCS1:** *I came because the job was hard, it was very hard in my homeland. The country here (BRAZIL) was in a very good phase and they told me I could work. So we came here to change (life), to make money and help the family too. We came to have a better life.* (E1, E2, E3, E8, E10, E11, E32)

### Central Idea B - War

**DCS2:** *I came because there was a civil war in my country and then my father took us out. Things there weren’t good, there were no conditions.* (E5, E6, E9, E23, E28)

### Central Idea C - Violence

**DCS3:** *I ran away and left my children because I didn’t have the money to take them with me. I ran away from thieves! I was robbed! They entered my house at night, raped me, took everything! I recognized one of them and then they warned me: if you go to the police, we will kill you. I got chased! Things got tough! Then I ran away from my country.* (E4, E21, E26, E31)

### Central Idea D - Adventure

**DCS4:** *I wanted to start traveling to other countries. I always saw Brazil on television and thought it was beautiful. I like Brazil, I would like to have been born here, I really wanted to come here. Then when I decided to leave my country, it was a take it or leave it moment and so I left.* (E14, E29, E30, E33)

### Central Idea E - Family Problems

**DCS5:** *I came because of family problems... My stepfather, my grandfather, my aunt, family issues. I don’t like to tell this story. I came to be away from the family.* (E13, E20, E27)

### Central Idea F - Study and Religion

**DCS6:** *I always wanted to go to college outside my country, at the same time I wanted it to be a course on my religion. I had a tight heart feeling, I didn’t want to get into trouble. I got in touch with several places, but I had not idealized it, suddenly I had this option of being able to come and study in Brazil.* (E15, E22, E18)

### Central Idea G - Political Issues

**DCS7:** *I came as a refugee, me and my family. In my country many things happened... political issues. The chase made us run away.* (E12, E24)

### Central Idea H - Health

**DCS8:** *My pregnancy wasn’t running well, the doctor provided the papers...* (E7)

### Central Idea I - Family Decision

**DCS:** *It wasn’t me who chose it, it was my father and then... I never asked why he chose Brazil.* (E17)

### Central Idea J - Study

**DCS9:** *I’ve been here for 5 years. I arrived to take an undergraduate degree. I took my undergraduate course and ended up staying to try a master’s degree.* (E19)

### Central Idea K - Family Reunification

**DCS10:** *My husband is from here.* (E25)

### Central Idea L - Did Not Answer (E16)

Among the Central Ideas related to local reception, two contradictory conceptions stood out: Feels discriminated against due to racism (45.5%) and Feels embraced (42.4%). Other ideas also popped up: Does not feel embraced (3.0%), Feels tolerated (3.0%), and Evasive (6.1%).

### Central Idea A - Feels Discriminated Due to Racism

**DCS1:** *It’s not easy... there’s some unusualness... The problem is that we are black people of dark skin and there is also xenophobia. That’s too strong! They let you enter the country and then they don’t embrace you and don’t treat you well. On the street, they don’t know you, but they look at you with indifference, distrustfully. When you look for a job, they separate you. The owner of a day care center hired me to help with the care of the children and made me work during the renovation of the place. I had to take tile packs upstairs. A colleague felt sorry for me, came to help me, couldn’t take it and fainted! At school, when we study, we are always chosen last to join groups, as if we weren’t smart. On the bus, on the subway, when we sit down, people get up, they also push you. We are mistreated, they call us names, they call our children monkeys. We stick to our goal. People here are really racist! It’s a lot of racism, a lot of discrimination, that’s too bad!* (E1, E4, E5, E6, E12, E14, E15, E18, E19, E21, E22, E24, E27, E28, E29)

### Central Idea B - Feel Embraced

**DCS2:** *I really liked the way they received me here in Brazil. I met good, loving, and friendly people. I had a child in my belly, I gave birth to my child here. I think Brazilians take good care of people, I’m treated very well! I see no difference between us and Brazilians. Brazil is good!* (E2, E3, E8, E9, E10, E11, E13, E17, E20, E23, E25, E30, E31, E33)

### Central Idea C - Does Not Feel Embraced

**DCS3:** *You don’t fit here, you can’t stay here!* (E16)

### Central Idea D - Feels Tolerated

**DCS4:** *They treat me well... They say good morning, nothing more than that...* (E7)

### Central Idea E - Evasive (E26, E32)

**DCS5:** *I don’t know... I don’t know if it’s good or bad, I don’t know anything... I’m not fine here.*

With regard to the presence of post-immigration illness, there were 3 Central Ideas: Did not get sick (51.2%), Got sick (45.4%), and Arrived sick (3.4%). The Central Ideas Got sick identified the following types of diseases: Emotional Problems (27.2%), Physical and Emotional Problems (9.0%), Physical Problems (6.1%), Not Specified (3.1%). In addition, there is the Central Idea Arrived sick (3.4%).

### Central Idea A - Did Not Get Sick

**DCS1:** *My health remained the same, the move didn’t bring any problems... I know it was difficult at first, I was sad... I know I can’t be nervous, I prefer to be at my place, be who I am. It’s just nostalgia, but I don’t have problems, I’m in good health.* (E4, E7, E8, E9, E11, E13, E16, E17, E20, E21, E22, E25, E27, E30, E31, E32, E33)

### Central Idea B - Got Sick Emotional Problems

**DCS2:** *Tension...a lot of tension. I left my family, my parents, there (in my country) I was strong. I think of my daughter who until now I don’t even know if she’s alive or dead... It hurts so much! It messed with my health and my heart... I’m sick in my mind and heart... and this country makes people sick. The doctor said I suffer from depression... it must be true.* (E3, E5, E6, E14, E19, E23, E24, E28, E29)

### Central Idea C - Got Sick Physical and Emotional Problems

**DCS3:** *We go through a lot. Everything was disturbing me, reflecting in my stomach. I was hospitalized, I had high blood pressure, but I’ll move on.* (E12, E15, E18)

### Central Idea D - Got Sick Physical Problems

**DCS4:** It got difficult, I have migraines and, as it is very hot in my country, here I have pain in my bones. (E1, E10)

### Central Idea E - Did Not Specify the Problem

**DCS5:** *My health is bad...* (E2)

### Central Idea F - Arrived Sick

**DCS6:** *It wasn’t okay. When I got here I was sick... tension.* (E26)

With regard to the project of staying in Brazil, the following Central Ideas emerged: Decided to stay (42.5%), Will not stay (27.3%), Undecided, (18.2%), Stay in case they get a job (9.0%), and Fearless (3.0%).

### Central Idea A - Decided to Stay in Brazil

**DCS1:** *I’m staying in Brazil. I have no way to return, I ran away! I even managed to visit my family, but no... My son was born here. Despite the conditions and difficulties, I will continue here, persist, and make my dreams come true.* (E2, E3, E4, E8, E16, E20, E21, E23, E24, E26, E27, E28, E29, E30)

### Central Idea B - Will Not Stay

**DCS2:** *I can’t continue. I’ll spend some time, gain experience and then I’ll go somewhere else. I’ll stay until things get better. God willing, one day I’ll go back to my country, make a contribution to my parents. It will be safer and more peaceful.* (E5, E7, E12, E13, E14, E15, E17, E18, E19)

### Central Idea C - Undecided

**DCS3:** *I don’t know whether to stay here or go somewhere else... I came for my husband and he wants to come back. In fact, I don’t know about tomorrow, only time...* (E9, E10, E11, E22, E25, E33)

### Central Idea D - Stay in Case they Get a Job

**DCS4:** The most important thing is to find work, a job. Then I want to bring my children and we stay here. But, if I don’t get a job, if the job is only for Brazilians, I’ll go back to my country. (E1, E6, E32)

### Central Idea E - Fearless

**DCS5:** *I’m hanging around, it will depend... If it’s not good, I might even go back after four or five years...* (E31)

## DISCUSSION

### Who they are, Country of Origin, and Current Life Situation

More than half of the women (60.6%) were between 31 and 40 years old. The findings, average age of 31.2 years, are not far from the average of 33.6 years evidenced in a study on Haitian immigration^(9)^. The numbers highlight a productive age group, a considerable factor for the formulation of strategies, policies, and socio-labor actions aimed at incorporating the group into the community.

Almost seventy percent of the immigrants (69.7%) came from Angola. Civil conflicts and agreements in the area of education may have driven women’s search for the Brazilian land. Trade relations between the two countries and the absence of language barriers^(6)^ also contribute to the process.

Political and environmental problems, with emphasis on a major hurricane, contributed to the Haitian exodus. Brazil, due to the social actions developed in Haiti, as well as the grant of humanitarian visas, has become one of the choices for immigration^(10)^, even in the face of initial difficulties with the language. At present, Haiti is a socioeconomically shattered country and is thus experiencing the emergence and progression of cholera and aids epidemics.

Immigrants from Congo and Ivory Coast, countries in ­sub-Saharan Africa, south of the Great Desert, were ­identified. European colonization reached these countries late, imposing cultural and linguistic orientations and setting up borders. Currently, rich countries such as China create dependencies and debts to the region^(11)^.

The Democratic Republic of Congo stands out for its essential mineral wealth for the electronics industry. However, ethnic and political conflicts contributed to the departure of millions of people and many became refugees. Rape, as a weapon of war against women, consolidated the country as the worst place in the world for this group. The cultural affinity, the impossibility of receiving a visa in another country, in addition to agreements for studies, motivated their coming^(12)^.

In Ivory Coast, civil war and ethnic conflicts make life difficult for the population. France, even after the colonial period, tries to maintain its protagonism in the place. The situation contributes to the flight of the female population. Ethnic and social identities, based on cultural roots, contribute to Brazil being one of the destinations for Ivorian immigrants^(13)^.

The situations mentioned confirm sociopolitical and environmental causes as factors that positively impact the exodus of black women from their countries of origin.

As for the level of education, more than half of the interviewees (55.4%) finished high school. The best educational indexes were with the Angolan women, who formed the largest number of participants in the sample. Even with different sample numbers, the finding was consistent with research carried out in Florianópolis^(14)^.

It is important to clarify that the black population represents more than 50% of the Brazilian society and is exposed to multiple weaknesses. It is out of the job market^(15)^ and in ­non-managerial positions, opening up the segregation present in the country. Black immigrants become part of this population group, making the situation even more exuberant.

The immigrants reported that, when they get a job, they are subject to various types of injustice. Employability is incompatible with the skills of workers and, even when they have higher levels of education, they face obstacles in obtaining jobs with social guarantees and earnings consistent with those of the market^(14)^. One interviewee did not answer the question, an aspect that may attest shame or fear.

### Reasons for Coming

The reasons that brought immigrants to Brazil show the aspects that weave mobility today and highlight the different reasons for immigrating.

The “Economic issue” was the most powerful determinant for immigration and it is important to clarify that Brazil, in recent times, has stood out as an important player in the global financial scenario. Repeatedly, the search for better living conditions is evidenced by the conjunctural contrasts between the countries of origin and destination^(16)^. It is also noted that in terms of gender, economic resources, and immigration, women are in a worse position.

Mobility related to “War” distinguishes immigration as a path associated with family protection and survival itself ^(16)^. Civil, religious, and ethnic conflicts sometimes precipitate a belligerent state that ends up expelling the natives. Especially on the African continent, such events have been a constant.

“Violence” is one of the most potent causes of female immigration. In this construct, rape, as a weapon of war^(17)^, and aggressive actions establish dread, fear, which often silence the subject. Leaving everything behind, including children, is not an uncommon situation.

“Adventure” reveals that women have been gaining more autonomy in their life choices and that they do not always migrate due to expulsion issues. Female migratory experiences have peculiarities associated with gender, life history, and age group^(18)^. They are searches for new ways of living.

“Running away from family problems” points to migration as a tool for reconfiguring the subject’s life inside and outside the group of origin. Moving away from the family base makes it possible for old issues to be supplanted and new relationships to be developed^(19)^.

“Study and religion” shows that studying in conditions harmonized with personal needs is one of the justifications for immigration^(17)^. Some traditional denominations settled in Brazil added an educational system consistent with the professed dogmas, establishing a relationship between geographic mobility and devotional education.

“Political issues” account for a significant number of displacements. These are clashes resulting in the movement of subjects, when many are compelled to move to protect their lives and that of their families: they are the refugees. The construct has been a strong pretext for African immigration.

Regarding “Health”, the Universal Declaration of Human Rights recommends attention to all individuals. In Brazil, one of the pillars of the Brazilian Public Health System (*SUS*) is the principle of universality, a concept that includes all individuals, even foreigners, who can have their needs met. Therefore, the movement to take care of health responds to the specific needs of the immigrant.

The “Family decision” refers to obedience to norms and rules, common attributes in patriarchal family systems, which contributes to the selection of a place to live being accepted as reliable. The migratory journey of women accompanying their families of origin is usually associated with their stage of life.

Individuals who migrate for “Study” aim at better qualifications and, consequently, a better life^(20)^. Discovering new cultures and advancing in academic life helps students to enjoy this immigration experience. International exchanges foster these possibilities.

“Family reunification” encourages human movement. A significant portion of women immigrate to be reunited with relatives. In this category, nuptiality is included, which emphasizes the reconstruction of the path of life from a different place.

“Did not answer” is an important discursive category, since silence is a way of communication, a way of replying. The unsaid is a meaning constructed by the interviewee, it is the significant silence, which expresses discomfort. Not speaking can illustrate affections, such as memories of people and places, in short, nostalgia.

The reasons for moving to another country reported by the women participating in the study are varied. However, regardless of the reasons, this process requires care and attention.

### Embracement in the Country

In Brazil, restrictive measures associated with the arrival of non-hegemonic groups, that is, non-whites, are differentiated^(17)^. Thus, knowing how the country receives black immigrants was considered relevant.

“Feeling discriminated against due to racism” shows that the traveler’s color, ethnicity, and social capital are decisive in the way of embracing. Different groups agree that dark-skinned people are inferior and that contextual disadvantages are a consequence of this circumstance^(5)^. In Brazil, the hegemony of white individuals is configured as a social and economic force; such a conception dialogues with a substantial segment of the society.

The fact that the first Africans arrived in Brazil as slaves fueled the idea of inferiority, legitimizing racism. It should also be noted that for a portion of the population, black immigrants are seen as a threat, as outlaws who disturb order.

“Feeling embraced” dialogues with a structuring bias of the Brazilian people. The colonial and post-colonial period marked the Brazilian population formation, made up of individuals from different places. This characteristic confirmed the country as a hospitable place for travelers and immigrants^(21)^, giving rise to the conception of Brazilian racial cordiality. This elaboration invites individuals from “risk nations” to start over in life, even if it is different for black immigrants.

In more recent times, the false idea of harmonious multiracial coexistence has been demystified. The narrative of peaceful coexistence distinguishes a powerful denial of racism in hegemonic groups and manifests a collectivity that is not used to discussion. Part of the immigrants can absorb this *truth*, apprehending the discourse of racial equality in the country^(22)^.

The “Do not feel embraced” shows that, for many Brazilians, immigrants are not welcome^(5)^. Appearance, culture, and economic status all compete to distance immigrants from natives. Interaction between groups constitutes a form of welcoming; sheltering the other facilitates immigrants social inclusion.

“Feeling tolerated” highlights that sectors of society show a conservative attitude towards immigrants, especially those from underdeveloped countries^(5)^. Coexistence is supported, which encourages the arrival of immigrants. In this construct, on several occasions, the individual is not subjectified, but observed only as a cog in the work machine.

The “Evasive” category marks a void, highlights dismay. The change of place of origin translates into a category of exile, an uprooting. Being a foreigner means not being a native of the country you live in, it means being a non-citizen; it is a circumstance that brings out losses and the feeling of non-belonging.

### Health and Immigration Process

The migratory situation^(2)^ is crossed by different conditions that can compete to become risks to the health of the mobilized. It is mentioned that the health system, in different circumstances, is not prepared to receive immigrants. Language barriers and the generalist view of “immigrants” exclude experiences and epidemiological issues. There is a gap in transdisciplinarity between the health sciences and the social sciences, a factor that aggravates the distance between the service and the migrant.

The women who “did not get sick” reinforce the idea that immigrants can present better health status and be resilient in the face of challenges^(2)^. Disorders associated with acculturation, which is part of the adaptive process, are usually observed. The migratory route, by itself, may not be the fundamental factor for the illness of those involved, but part of the interviewees report initial difficulties and the choice for isolation. Healthy female immigrants tend to weave networks of reciprocity and solidarity among peers.

The “Emotional Problems” attest that the migratory process entails ruptures, it is a context that involves legal and affective issues. Family, social, cultural, and political outcomes can elicit the immigrant syndrome. They are painful and traumatic absences. Many immigrants also experience maternal care in situations of solitude. Moreover, some women unable to take their children experience the transfer of care, known as transnational mothering. These conditions can progress to depression. Cultural psychology has called the depression that affects African immigrants black depression^(23)^.

“Physical and emotional problems” are the result of a set of situations. When leaving their homeland, new feelings emerge in the traveler’s life: the longing for what was left and the uncertainty of the future start to compose their daily lives^(24)^. They bear the stress of acculturation, the struggle for survival, loneliness, and fear of failure. The selection of the place and/or shelter and socioeconomic conditions can strengthen a scenario that impacts health. Identity reconfigurations and re-elaborations contribute to instigating disturbances, malaise, and various symptoms; sometimes it is an unhealthy complex.

“Physical problems” are associated with climate and environmental variability. Added to this are the social and political circumstances that determined the move, a framework that can favor the situation of illness^(2)^. Among the main complaints are: fatigue, musculoskeletal problems and headache, symptoms that make up the diagnosis of Ulysses Syndrome, a condition caused by travelers’ everyday tensions and living conditions. The pathology embodies a migratory mourning and the responses to the condition are related to the individual’s resilience.

The category “Did not specify the problem” denotes that the entanglements of adapting to the new foster questions that are difficult to be described or detailed; they are complex dysfunctionalities^(17)^. They cover inadequate housing conditions, precarious socioeconomic situation, and incidents at work. Immigrants are “obliged” to rebuild their lives, impelled to fulfill new roles and tasks to survive in a stressful environment. Part of the group may not have internal tools to overcome all barriers, a factor that affects the individual’s health.

The relationship with migratory characteristics, legality, or otherwise of the subject in the new country, in addition to internal adaptive devices influence health and quality of life. The statement “Arrived sick” reveals that leaving part of life behind and recognizing the chances of future obstacles pave the way for illness^(24)^. It is important to consider that immigrants may be sick because they brought diseases from their country of origin, which would imply social and even economic risk. The situation of vulnerability accompanies the arrival of many immigrants, with tension being one of the expressions of the anguish and suffering experienced.

### Project of Staying in the Country

The departure and entry of individuals in the context of migration change the perspectives of the country of origin and of reception, bringing about significant population changes. The social, health, and economic panorama is affected.

The statement “Decided to stay” shows, in line with a previous study, that black immigrant women want to consolidate a new life in the country^(25)^. It should be noted that there are few returns among those who immigrated due to wars or political issues. They are women who adjust family and affective networks to start over.

The statement “Will not stay” transits between return immigration^(25)^ and/or voluntary repatriation. Many immigrants move to resolve urgent issues, such as those of a military and economic nature. These are experiences based on changes, achievements, and reintegration in the country of origin. Some individuals already arrive with an elaborate return project.

The statements in the “Undecided” category are marked by transience. The group assumes a debate between permanence and return, the stay depending on the conditions offered by the new country. Different situations influence decision-making: affective, economic, and sociocultural issues. Although they have a life reconstruction project, part of the immigrants link their stay to future events.

“Permanence conditioned to employment” is in tune with a Brazil that is open to receiving immigrants and signals potential for work. Simultaneously, it is evident that the labor insertion of immigrants sometimes happens in degrading situations. These are services that do not require qualification, nor do they offer an employment relationship. The scenario distinguishes migrants as part of a labor market, which contributes to the formation of an irregular economy chain, which hydrates the businessmen’s wealth^(24)^.

Contemporary migrations highlight individuals who fall into the category “Fearless”, that is, those who experience life in different places. Along the way, they get to know new people and cultures, they are mobile immigrants^(23)^. They travel without a definitive project, looking for a place that meets their existential demands.

## CONCLUSION

The study points out that black immigrant women are adults, of working age, who live in extremely difficult conditions.

The reasons for immigration respond to the search for better living conditions: economic issues, flight from wars, and the possibility of completing the studies, among others. As for the perception of embracement, two antagonistic points prevail: being well received and being rejected due to racism, highlighting the strong distinctions existing in the country. The interviewees’ health crosses the field that circumscribes immigration and points to the importance of specific actions.

Origin, ethnicity, socioeconomic profile and capital can enhance vulnerabilities. The pilgrim’s journey from the origin to the arrival at the destination traverses paths highlighted by adversity.

There are still few studies addressing the gap between the emigrant and the immigrant, a dismantling of the puzzle that constitutes the subject.

Human mobility can form scarred tessitures; therefore, it is necessary to think about procedures to lessen the consequences. Thus, efforts on the part of the receiving country are essential, since the article points to reports of living conditions that exuberantly expose patterns related to the group of black immigrant women.

The need to organize an internal agenda to serve similar groups is acknowledged, an essential attribution to the nation that aims to receive people, a commitment that refers to the promotion of means to embrace, aggregate, and incorporate people as citizens.
